# Differences in the ability to suppress interferon β production between allele A and allele B NS1 proteins from H10 influenza A viruses

**DOI:** 10.1186/1743-422X-7-376

**Published:** 2010-12-31

**Authors:** Siamak Zohari, Muhammad Munir, Giorgi Metreveli, Sándor Belák, Mikael Berg

**Affiliations:** 1 Swedish University of Agricultural Sciences (SLU), Department of Biomedical Sciences and Veterinary Public Health, Section of Virology, SLU, Ulls väg 2B, SE-751 89 Uppsala, Sweden; 2 Department of Virology, Immunobiology and Parasitology, National Veterinary Institute (SVA), Ulls väg 2B, SE-751 89 Uppsala, Sweden

## Abstract

**Background:**

In our previous study concerning the genetic relationship among H10 avian influenza viruses with different pathogenicity in mink (*Mustela vison*), we found that these differences were related to amino acid variations in the NS1 protein. In this study, we extend our previous work to further investigate the effect of the NS1 from different gene pools on type I IFN promoter activity, the production of IFN-β, as well as the expression of the IFN-β mRNA in response to poly I:C.

**Results:**

Using a model system, we first demonstrated that NS1 from A/mink/Sweden/84 (H10N4) (allele A) could suppress an interferon-stimulated response element (ISRE) reporter system to about 85%. The other NS1 (allele B), from A/chicken/Germany/N/49 (H10N7), was also able to suppress the reporter system, but only to about 20%. The differences in the abilities of the two NS1s from different alleles to suppress the ISRE reporter system were clearly reflected by the protein and mRNA expressions of IFN-β as shown by ELISA and RT-PCR assays.

**Conclusions:**

These studies reveal that different non-structural protein 1 (NS1) of influenza viruses, one from allele A and another from allele B, show different abilities to suppress the type I interferon β expression. It has been hypothesised that some of the differences in the different abilities of the alleles to suppress ISRE were because of the interactions and inhibitions at later stages from the IFN receptor, such as the JAK/STAT pathway. This might reflect the additional effects of the immune evasion potential of different NS1s.

## Background

Type I interferons (IFNs) play an essential role in both the innate immune response and the induction of adaptive immunity against viral infections. Viral infections trigger the production of type I IFNs (IFN-α/β) [1,2], which leads to the activation of several hundred IFN-stimulated genes (ISGs). These genes encode a variety of antiviral proteins and cytokines, leading to the protection of the host from further viral infections [3,4].

The main viral sensors in most mammalian nucleated cells are RNA helicases, retinoic acid-inducible gene I (RIG-I) and melanoma differentiation-associated protein 5 (MDA-5), which recognises viral single-stranded RNA (ssRNA) and double-stranded RNA (dsRNA) [1,5-9]. Many cells also recognise viral dsRNA through Toll-like receptor 3 (TLR3) [1,10]. The binding of virus-derived nucleic acids to RIG-I, MDA-5 or TLR3 results in a coordinated activation of the transcription factors nuclear factor kappa B (NF-κB) and interferon regulatory factor 3 (IRF-3), leading to IFN-β production in mammals [6,7,10].

Although a variety of cellular signalling has been evolved in host cells for detecting and responding to viral infection, most viruses possess mechanisms to evade these host immune responses to various degrees [7,11]. For example, many viruses have developed a multitude of mechanisms to evade the IFN response by either blocking IFN synthesis or interfering with the functions of IFN [12].

In the case of influenza A viruses, the non-structural gene (NS) has been shown to be responsible for viral anti-IFN activities [13-16]. The NS gene of influenza A viruses encodes for two proteins [17]. The first is through the translation of unspliced mRNA, which encodes a protein of 26 kDa known as non-structural protein 1 (NS1). The second is a 14 kDa nuclear export protein (NEP, formerly called NS2) translated from spliced mRNA [18].

The NS1 protein antagonises both the induction of IFN-β [19,20] and the activity of several IFN-induced proteins with antiviral activities such as protein kinase R (PKR) and 2'-5'oligoadenylate synthetase (OAS) [21-23].

The NS gene can be classified into separate gene pools, termed alleles A and B [24,25]. Between allele A and B, 63-68% nucleotide identity and 66-70% amino acid identity was found between the NS1 proteins. The NS allele A is more common and is the only subtype found in mammalian-adapted isolates. In a comparison between amino acid sequence of avian allele A and B viruses with an amino acid sequence of human viruses, six amino acid motifs, or signatures, were found between human and avian allele A viruses, and 35 signatures between human and allele B viruses, indicating that allele B viruses are more distinct from mammalian origin viruses [26]. This suggests that the adaptation of NS1 plays an important role in the pathogenicity of avian influenza viruses in mammalian species.

In our previous study concerning the genetic relationship among H10 avian influenza viruses with different pathogenicity in mink (*Mustela vison*), we found that these differences were related to amino acid variations in the NS1 protein. We demonstrated that in a model system using polyinosinic-polycytidylic acid (poly I:C)-stimulated mink lung cells, the NS1 protein of influenza A virus isolated from mink (A/mink/Sweden/84 (H10N4)) down regulated type I IFN promoter activity to a greater extent than the NS1 protein of prototype H10 virus (known as virus/N (A/chicken/Germany/N/49 (H10N7)) [27].

In this study, we extend our previous work to further investigate the effect of the NS1 from different gene pools on type I IFN promoter activity, the production of IFN-β, as well as the expression of the IFN-β mRNA in response to poly I:C.

## Results

### Activation of IFN-β promoter

**Figure 1:**
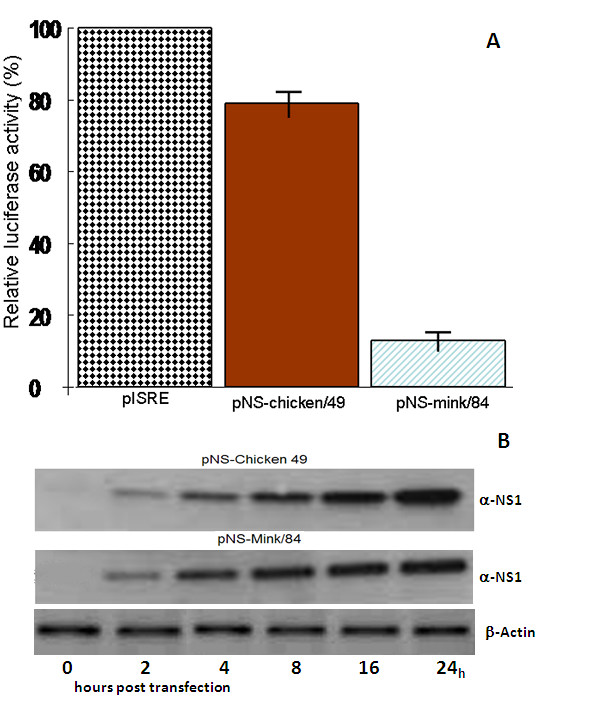
**Prevention of poly (I:C) induced activation of an IFN-β promoter by the NS1 protein in A549 cells**. (A) Forty-eight hours after transfection, the cells were harvested and assayed for luciferase activity. Average relative luciferase activities are reported. Data are expressed as the mean ± S.E. for the three independent experiments performed in duplicate. (B)Western blotting was performed to compare level of expression of the two NS1 constructs. Expression of the NS1 proteins in A549 cells transfected with the NS1 constructs; pNS-chicken/49 and pNS-mink/84, was confirmed at 0, 2, 4, 8, 16 and 24 hours post transfection.

First, we studied the ability of NS1 from "mink/84" and "chicken/49" to inhibit the induction of transcription of the IFN-β gene, using the model system ISRE-Luciferase and Poly I:C stimulation. This reporter system relies on expression of IFN and the subsequent signalling from the IFN-α/β receptor leading to expression from the ISRE reporter gene (luciferase). Although both NS1 from "mink/84" and "chicken/49" showed a significant suppressive effect on the luciferase activity, it was considerably stronger in cells transfected with "mink/84" with an average of 6.8 fold decrease (85.3%) in A549 cells (Figure 1A), compared with "chicken/49", that on average produced a 20.8% decrease in A549 cells.

### Expression of NS1 proteins in A549 cells

To find out whether the difference in inhibition of IFN-β promoter is duo to difference in- or insufficient expression of the NS1 proteins in A549 cells, the level of expressed NS1 proteins was confirmed by western blot analysis. The cells were lysed at 0, 2, 4, 8, 16 and 24 hours post transfection and western blotting was performed. The NS1 proteins from both constructs were expressed in high quantity and the level of allele A NS1 was comparable to NS1 protein of allele B (Figure 1B). The western blotting showed that the expressed protein from both "mink/84" and "chicken/49" was homogenously accumulated in A549 cells and there was no notable difference between alleles in term of NS1 production (Figure 1B). Thus, the results indicated that the difference in IFN-β induction in the presence of allele B NS1 protein was not due to difference in allele B NS1 protein expression and accumulation in the cells.

At this point it was not clear if this result corresponded to differences in the ability to downregulate IFN production, or that the signalling pathway leading to ISRE transcription is influenced, or both. To sort out this, IFN protein production was measured by an ELISA.

### IFN-β production

**Figure 2:**
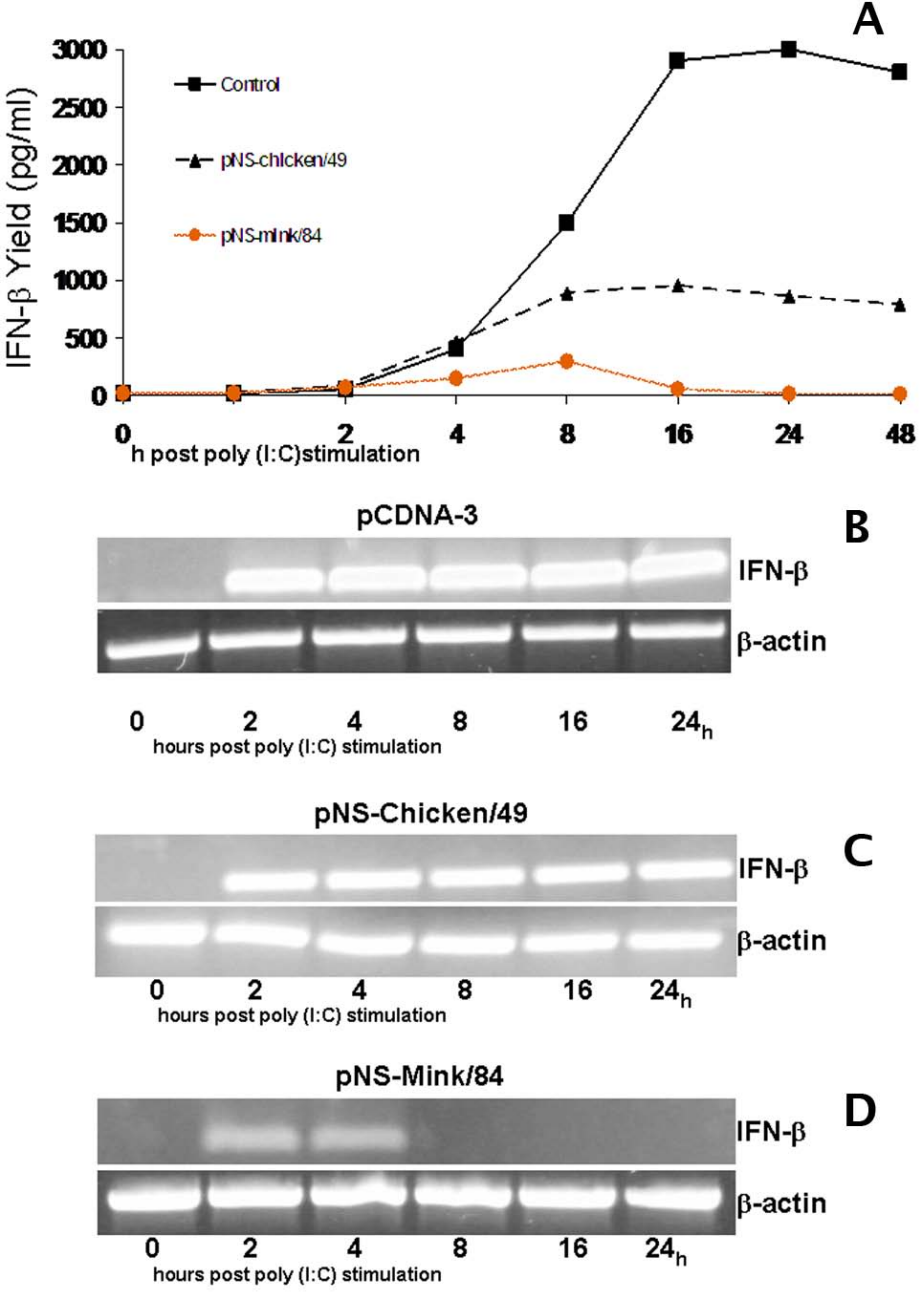
**IFN-β release in the supernatant and expression of IFN-β m-RNA in human A549 epithelial cells in response to poly I:C challenge at the presence of different NS1 proteins**. (A) The concentration of IFN-β in A549 cell supernatants was assayed. Cell were transfected with plasmids containing either the NS gene of "mink/84" or "chicken/49" virus or was mock treated, 24 hours later cells were stimulated with 5 μg/ml of poly I:C. The cell supernatants were collected at 0, 2, 4, 8, 16, 24 and 48 hours post-poly I:C stimulations. Expression of IFN-β m-RNA in A549 cells in response to poly I:C challenge at the presence of different NS1 proteins. A549 cells were transfected with (B) empty pCDNA-3 vector, (C) pNS-mink/84 and (D) pNS-chicken/49 respectively, 24 h later cell were treated with 5 μg/ml poly (I:C) for indicated time. Data are expressed as the mean value for the three independent experiments performed in duplicate.

The IFN-β protein was detected in the cell medium of the control cells after a lag of 2 to 4 hours after poly I:C stimulation, followed by the linear accumulation of IFN-β in the cell culture supernatant. The peak yields for control cells were reached about 16 to 24 hours post-stimulation (Figure 2A). Although low levels of IFN-β were secreted by cells transfected with different NS1s, significant differences were observed between these NS1s. Those cells expressing the NS1 protein of "mink/84" virus were weak producers of IFN-β, with at least 10 times lower levels of IFN-β secreted in the cell culture supernatant than the control cells. In these cells IFN-β secreted to the supernatant reached the maximum yield 8 hours post-stimulation and declined rapidly to a low level for the rest of the experiment. By contrast, cells expressing the NS1 protein of "chicken/49" were better producers of IFN-β with the profile lower but similar to that observed with the control cells (Figure 2A). This indicates that NS1, in this system, suppresses IFN protein production rather than the signalling from the IFN receptor.

### Expression of IFN-β in response to poly I:C

To determine whether the reduction of IFN-β production was caused by the suppression of the expression of the IFN-β gene, we compared gene expression kinetics in A549 cells stimulated with poly I:C in the presence or absence of different NS1 proteins.

In the control cells, IFN-β mRNA was detected in increased amounts during the entire period of the experiment (Figure 2B). The same profile was observed in the cells expressing the NS gene of "chicken/49 " (Figure 2C). Transcript levels in the control cells were significantly increased 2 to 4 hours post-stimulation, reaching a plateau at the end of the experiment. Four hours after stimulation, the NS1 protein of the "mink/84" effectively suppressed IFN-β gene transcription in A549 cells (Figure 2D). The activation of the IFN-β gene expression in cells transfected with plasmids carrying the NS gene of "chicken/49" resulted in increased levels of IFN-β mRNA showing the same trend similar to the control cells.

The RT-PCR analysis of the INF-β mRNA presented in the stimulated A549 cells expressing NS1 of "mink/84" or "chicken/49" confirmed that the NS1 protein of "mink/84" effectively suppressed IFN-β gene transcription in A549 cells, indicating that the main target of the "mink/84" NS1 is the induction of IFN.

## Discussion

One of the main strategies of the influenza A viruses to avoid host immune responses is to inhibit IFN-α/β expression or signalling to the neighbouring cells, which induce their antiviral state by the stimulation of transcription from the ISRE promoter-containing genes [28]. The viral NS1 of influenza A viruses is known to be an important regulator of innate immunity on many levels [13-16]. The NS1 inhibits host immune responses through two functional domains: an N-terminal RNA binding domain and a C-terminal effector domain [19]. The effector domain interacts with proteins involved in the 3'-end cellular mRNA processing, inhibits mRNA export and pre-mRNA splicing of host cell transcripts and interacts with components of the nuclear pore complex as well as the mRNA export machinery [29-34]. The N-terminal RNA binding domain binds to both single- and double-stranded RNA that might inhibit the activation and/or signalling of antiviral proteins, such as RIG-I, PKR, OAS/RNase L, activators of mitogen-activated protein kinase and transcription factors involved in type I IFN and inflammatory cytokine signalling [20,22,23,35-37].

**Figure 3:**
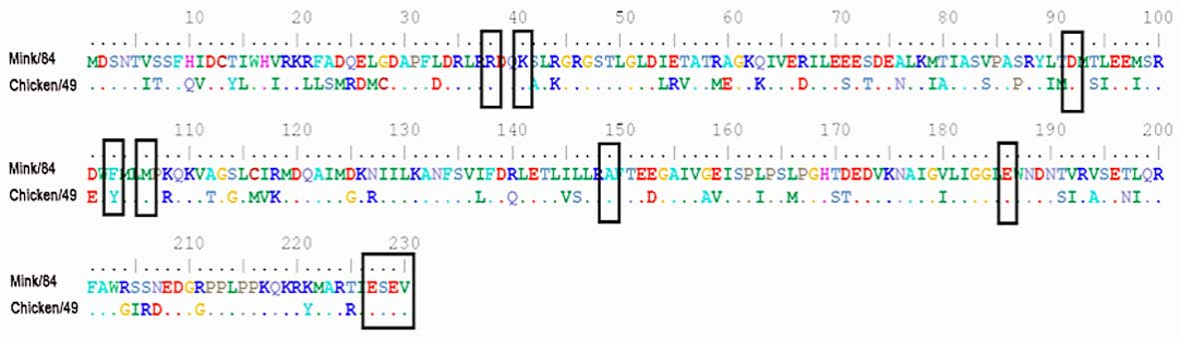
**The predicted NS1 amino acid sequence alignments for the "mink/84" and "chicken/49" viruses**. The boxes indicates the previously identified important amino acid residues for the function of NS1 protein in the infected cells.

Our previous study indicated that the NS1 protein is a potential key factor for the different pathogenicity levels of the H10 avian influenza viruses in mink (*Mustela vison*) [27]. In this study, we applied an expression plasmid system carrying the ORF of NS1 of two avian influenza viruses, showing the difference in pathogenicity in mink [38]. Furthermore, these viruses represent different NS alleles, one from A ("mink/84") and the other one from B ("chicken/49"). A comparison of the predicted amino acid sequences of the two NS1 proteins showed 71 amino acid differences (Figure 3). However, the two NS1 proteins were found to be very similar regarding the previously identified important amino acid residues for the function of NS1 protein in the infected cells [23,29,30,34,39,40].

Notably, the only difference was found in the site important for the NS1 protein's interaction with the 30 kDa subunit of cleavage and polyadenylation specificity factor (CPSF30) [27]. The NS1 protein interaction with the CPSF30 inhibits 3'-end processing of cellular pre-mRNA [29,30,34]. This function is mediated by two distinct domains: one around residue 186 [30] and the other around residues 103 and 106 [41]. The NS1 protein of "mink/84" possessed the amino acid Glu186, Phe103 and Met106, whereas the NS1 protein of "chicken/49" possessed Tyr 103. A previous study [41] showed that mutations at the NS1 protein CPSF30 interaction sites dramatically changed the effect of the NS1 to control host gene expression.

Both "mink/84" and "chicken/49" NS1s had a negative effect on the activation of the ISRE promoter, as shown by the luciferase activity. But the reduction was much stronger in cells transfected with the "mink/84" NS1 plasmid with an average of 85.3% decrease in A549 cells (Figure 1A), whereas pNS-chicken/49 on average produced a 20.8% decrease in A549 cells. As this final product is dependent on both the induction of IFN and luciferase from the IFN receptor, the exact mechanism by which this interference is mediated through can be either by inhibiting IFN induction signals via RIG-I, MDA-5 or TRL-3, the processing of IFN mRNA, or the downstream effects via IFN receptor signalling or luciferase mRNA processing.

Several studies have indicated that the blocking of virus-induced IFN-β promoter activation is mediated by the N-terminal RNA binding domain of the NS1 protein [42-44]. The 71 amino acid differences between the two NS1 proteins will most likely result in differences on the three-dimensional structure of the NS1 protein that could affect the function of NS1 in the suppression of IFN-β promoter activation.

Since the induction of the IFN-β promoter is associated with the production of IFN-β, we next investigated the level of endogenous IFN-β mRNA and the amount of IFN-β secreted in the cell supernatant. It has been observed that the NS1 protein of "mink/84" but not "chicken/49" strongly suppressed the expression of the IFN-β gene and secretion of IFN-β in the cell culture supernatant. In the time course study using A549 cells stimulated with poly I:C, IFN-β production displayed three distinct phases. After an initial rapid increase it reached a peak and then declined to lower levels. The production of IFN-β by poly I:C stimulation in A549 cells displayed a 2- to 4-hours lag followed by a steady increase in the accumulation of secreted IFN-β in the cell culture media. Maximal yields were observed at 16 to 24 h post poly I:C stimulation (Figure 2A).

Similar observations were made when mRNA levels were measured. The expression during poly I:C stimulation revealed an early up regulation of IFN-β transcripts starting at or before 2 h with a peak at 18-24 h after stimulation. During the first 4 h post-stimulation, we observed an up regulation of IFN-β mRNA transcripts in A549 cells expressing the NS1 protein of "mink/84". Thereafter, the IFN-β gene transcription was strongly suppressed, whereas a high level of the IFN-β mRNA expression continued in A549 cells expressing NS1 protein of "chicken/49" (Figure 2B,C&2D).

Future experiments are required to investigate the exact molecular mechanism behind this observation. This may require the use of animal experiments and also includes tools like reverse genetics, genomics and proteomic tools that allows the analysis of many parameters involved in the complex interplay between the NS1 and the host innate immune machinery.

## Conclusions

All these observations indicate that different non-structural protein 1 (NS1) of influenza viruses, one from allele A and another from allele B, show different abilities to suppress the induction of IFN mRNA; however, the exact mechanism is unknown. The results also demonstrate that the production of an important cytokine, IFN-β is affected by the function of NS1 protein from different genetic gene pools.

It is possible that NS1 interacts with one of the inducing pathways, or both, or that the mRNA processing is blocked. The latter can be studied by investigating another inducible gene other than an IFN-dependent one.

## Methods

After establishing an assay protocol for different part of our study, both NS1 construct were tested in duplicate at three independent experiments (each experiment was set up separately and carried out on different days).

### Construction of expression plasmids

The NS1 open reading frames (ORF) of influenza A virus strains A/mink/Sweden/3900/84 ("mink/84") and A/chicken/Germany/N/49 ("chicken/49") were amplified using the primers NS1Kpn 5' (5'-ATTCGGTACCAGCAAAAGCAGGGTGACAAAG-3') and NS1XhoI 3' (5'-TACCCTCGATAGAAACAAGGGTGTTTTTTAT-3'). Twenty-five microliter PCR-mix contained 1xPlatinum Taq buffer (Invitrogen), 200 μM dNTP, 2.5 mM MgCl_2_, (Invitrogen) and 3 μl cDNA. Reactions were placed in a thermal cycler at 95°C for 2 min, then cycled 35 times between 95°C 20 sec, annealing at 58°C for 60 sec and elongation at 72°C for 90 sec and were finally kept at 8°C until later use.

The 690 bp PCR products were digested with *Kpn *and *Xho*I and cloned between the *Kpn *and *Xho*I sites of the mammalian expression vector pcDNA3.1 (Invitrogen, Carlsbad, CA, USA), creating pNS-mink/84 and pNS-chicken/49 plasmid respectively. The integrity of the plasmids was confirmed by sequencing.

### Cell culture and transfection experiments

A549 cells, a type II alveolar epithelial cell line from human adenocarcinoma, (ATCC, CCL 185) were cultured in Dulbecco's modified Eagle medium (DMEM) and supplemented with 10% FCS in a humidified atmosphere of 5% CO_2 _at 37°C.

Transcriptional activity was assayed in the A549 cells. Cells were co-transfected with plasmids containing either the NS gene of "mink/84" or "chicken/49" together with reporter plasmids driving expression of *Firefly *luciferase (pISRE-TA-Luc) (Invitrogen) under the control of the IFN-stimulated response element (ISRE). The pRen-Luc plasmid containing the *Renilla *luciferase gene (Invitrogen) was used as internal control. The activity of the reporter gene were standardised by the *Renilla *luciferase activity. The inhibitory effect in cells expressing the various NS1s was expressed in folds of luciferase activity.

The transfection of the plasmids was conducted with FuGENE 6 reagent (Roche Molecular Biochemicals, Indianapolis, IN) in six-well plates according to the manufacturer's instructions. Initial experiments were conducted to optimise the efficiency of the transfection protocol. The day before transfection, cells were collected and seeded into six-well plates at 1 × 10^5 ^cells per well to achieve 70-80% confluence on the day of transfection. Each transfection group consisted of six wells in which three were poly I:C stimulated and three mock treated. Stimulation of the cells with the poly I:C was performed 24 hours after transfection of the pcDNA3.1/NS1 plasmid through the addition of 5 μg/ml poly I:C mixed in 100 μl DMEM without serum. Twenty-four hours later, the cells were harvested according to the protocol for the luciferase assay kit (Stratagene, Heidelberg, Germany), using 300 μl lysis buffer for each well. Samples were kept on ice and centrifuged for 2 min at 14,000 × *g *to remove cell debris before measurement of the luciferase activity. Luciferase activities were measured using 20 μl of each sample according to the manufacturer's protocol.

### Western blot analysis

All the transfections for western blot analysis were performed following the same protocol as described above. Briefly, cells were washed and lysed at 0, 2, 4, 8, 16 and 24 hours post transfection using Bio-Plex cells lysis kit (Bio-Rad Laboratories, Hercules, CA) according to the manufacturer's instructions. After incubation for 20 min at 4°C and three times thawing-freezing steps at -70°C, the lysates were centrifuged at 4500 rpm for 20 min. Concentration and quality of the protein were measured using Nanodrop ND1000 (Nanodrop Technologies, Wilmington, DE.) and by SDS-polyacrylamide gel electrophoresis (SDS-PAGE) followed by Coomassie blue staining. A total of 50 μg of the cell lysate was separated bySDS-PAGE in Ready Gel J 7.5% (Bio-Rad) and then electronically transferred onto polyvinylidene difluoride (PVDF) membrane (GE Healthcare, Uppsala, Sweden). The membranes were incubated in blocking buffer (PBS, 2% (wt/vol) bovine serum albumin) at room temperature for one hour on slow agitation, the NS1and β-actin proteins were detected using anti-NS1 polyclonal, the NS1 antibodies was raised in goat against a peptide mapping near the C-terminus of influenza A NS1 (sc-17596, Santa Cruz Biothechnology, INC) and anti β-actin (Sigma-Aldrich, Stockholm, Sweden) , followed by incubation with primary antibodies diluted in TBS-2% BSA at 4°C overnight. After intensive washing with TBS (PBS, 0.2% Tween 20) membranes were incubated with horseradish peroxidase (HRP)-conjugated anti-goat secondary antibodies for the NS1 and anti-mouse secondary antibodies for the β-actin detection for two hours at room temperature on continuous agitation. The blots were developed by an ECL advance kit from GE Healthcare and visualized in ChemDoc XRS system from Bio-Rad with Quantity One^® ^software.

### Human IFN-β ELISA

The concentration of IFN-β in stimulated A549 cell supernatants was determined using a commercially available VeriKine™ human IFN-beta sandwich enzyme-linked immunosorbent assay (ELISA) kit (PBL interferon source, Piscataway, NJ, USA) according to the manufacturer's instructions. The cell supernatants were collected at 0, 2, 4, 8, 16, 24 and 48 hours post-poly I:C stimulations. Briefly, microtiter strips were incubated with 100 μl of IFN standards, blanks and samples. After one hour of incubation, the strips were washed and detection antibodies were added. After incubation and an additional washing step, streptavidin conjugated to horseradish peroxidase (HRP) was added, and the strips were incubated at room temperature for 1 hour. The strips were again washed before the addition of the tetramethyl benzidine (TMB) substrate solution, after which the strips were incubated for 15 min at room temperature in the dark. The reaction was terminated by the addition of stop solution, and the optical density of the wells was read at 450 nm using a microplate reader Multiscan EX (Thermo scientific, MA, USA). Values for the samples were compared to those for the standard curve and the amount of IFN-β was estimated from the standard curve.

### Analysis of IFN-β mRNA by RT-PCR

RT-PCR was used to study the level of IFN-β mRNA expression in Poly I:C-stimulated A549 cells. The housekeeping gene β-actin was used as a control. RT-PCR was performed using the following primer pairs specific to human IFN-β and β-actin mRNA: IFN-β forward 5' GGCCATGACCAACAAGTGTCTCCTCC 3' and reverse 5' ACAGGTTACCTCCGAAACTGAGCGC 3', resulting a product of 550 bp; and β-actin forward 5' TGGGTCAGAAGGACTCCTATG 3' and reverse 5' AGAAGAGCTATGAGCTGCCTG 3'. Twenty-five microliter PCR-mix contained 1xPlatinum Taq buffer (Invitrogen), 200 μM dNTP, 2.5 mM MgCl_2_, (Invitrogen) and 3 μl cDNA. Reactions were placed in a thermal cycler at 95°C for 2 min, then cycled 35 times between 95°C 20 sec, annealing at 63°C for 60 sec and elongation at 72°C for 90 sec and were finally kept at 8°C until later use.

A549 cells were seeded in six-well plates and transfected with either pNS-mink/84, pNS-chicken/49 or empty pcDNA 3.1 vector as described above. Cells were stimulated with 5 μg/ml poly I:C mixed in 100 μl DMEM without serum. Cells were harvested and RNA was extracted for RT-PCR assays at 0, 4, 8, 16 and 24 hours post-stimulation.

RNA was isolated using TRIzol Reagent (Invitrogen) according to the manufacturer's protocol. RNA was DNAse-treated and quantified and purity measured at OD_260/280 _using a Nanodrop ND1000 (Nanodrop Tec., Wilmington, DA, USA). All RNA samples had an OD_260/280 _ratio in water between 1.9 and 2.1. 2 μg RNA was used to make cDNA templates using Superscript II (Invitrogen) according to the manufacturer's instructions and oligo-dT primers (Invitrogen).

## Competing interests

The authors declare that they have no competing interests.

## Authors' contributions

SZ conceived and designed the study, organized protocol developments, performed the transfection-, real-time RT-PCR, western blotting and ELISA analyses, contributed to interpretation of data and wrote the manuscript. MM, organized protocol developments, contributed to the interpretation of the findings and revised the manuscript. GM , contributed to and revised the manuscript. SB contributed to conception, interpretation of data, and revised the manuscript. MB additionally contributed to the study design, contributed to conception, interpretation of data and revised the manuscript. All authors' have read and approved the final manuscript.
